# The effects of mother’s education on achieving exclusive breastfeeding in Indonesia

**DOI:** 10.1186/s12889-020-10018-7

**Published:** 2021-01-06

**Authors:** Agung Dwi Laksono, Ratna Dwi Wulandari, Mursyidul Ibad, Ina Kusrini

**Affiliations:** 1 National Institute of Health Research and Development, the Ministry of Health of the Republic of Indonesia, Jakarta, Indonesia; 2 Doctoral Program, Faculty of Public Health, University of Airlangga, Surabaya, Indonesia; 3 Faculty of Public Health, Universitas Airlangga, Surabaya, Indonesia; 4 Faculty of Health, Nadlatul Ulama University, Surabaya, Indonesia; 5 Unit of Health Research and Development Magelang, Ministry of Health, Center Java, Java, Indonesia

**Keywords:** Breastfeeding, Exclusive breastfeeding, Education level, Nutrition education, Health education

## Abstract

**Background:**

Even though the Indonesian government have set regulations for maintaining exclusive breastfeeding practices, the coverage remains low. The study aims to analyze the effects of mother’s education level on the coverage of exclusive breastfeeding in Indonesia.

**Methods:**

This study used data from the 2017 Nutrition Status Monitoring Survey. It covered data of 53,528 children under 5 years old (7–59 months) as the samples. Variables included exclusive breastfeeding status, mother’s education level, mother’s age, marital status, employment status, gender, residence, under five’s age and gender. A binary logistics regression was performed in the final test.

**Results:**

Mothers who graduated from elementary school were 1.167 times more likely to perform exclusive breastfeeding compared to mothers who never attended schools. Additionally, those who graduated from junior high school had 1.203 times possibilities to give exclusive breastfeeding compared to mothers without educational records. While, mothers who graduated from high school were 1.177 times more likely to perform exclusive breastfeeding compared to those without educational records. Mothers who graduated from tertiary education had 1.203 times more possibilities to perform exclusive breastfeeding compared to mothers who were never enrolled to schools. Other variables also became affecting predictors on exclusive breastfeeding, such as mother’s age, mother’s employment status, child’s age, and residence.

**Conclusions:**

The mother’s education level positively affects exclusive breastfeeding practice in Indonesia.

## Background

Breastfeeding is a common method of providing breast milk as food supply for infants and young children. It is the cheapest and simplest method to meet the baby’s nutritional needs. Breast milk will improve sensory and cognitive abilities and protect children from infectious and chronic diseases. Poor infant feeding practices may impact on children’s growth and development [1, 2]. A meta-analysis study used 17 previous studies that discover an increase in IQ scores of 3.44 points in breastfed children. Meanwhile, in the meta-regression, none of the studies explained the heterogeneity [3].

Exclusive breastfeeding (EBF) is a method of giving breast milk merely for infants to provide complete nutrition in the first 6 months of life [4]. Exclusive breastfeeding has many benefits for mothers and babies. For babies, EBF can reduce infant mortality due to common infectious diseases, help recovery, and increase children immunity. Regarding its benefits for mothers, EBF is a safe feeding method that can protect mothers from the risk of ovarian and brceast cancer and reduce obesity [5–7].

Although EBF has been proven to a positive a positive effect [3, 8, 9], the coverage of EBF is still very low. Globally, the coverage of EBF was 30–50% [1], but was around 35.7% in Indonesia [10]. Previous studies have identified intrinsic and extrinsic factors that affect EBF. Despite having regulations to maintain EBF practices (Law Number 36/2009 concerning Health), the coverage of EBF in Indonesia remains low, especially for uneducated and employed mothers [11].

The low coverage has something to do with traditional breastfeeding practices in native Indonesian tribes. Some native tribes introduce food or drinks to babies who are recently a few days old. The Gayo, Javanese, and Muyu tribes commonly feed their babies honey, sugar water, and sago solution [12–15].

Based on the background, this study aimed to analyze the effects of mother’s education on exclusive breastfeeding practice in Indonesia. The results of this study vitally become basic references for policymakers to formulate policies in improving the coverage of EBF in Indonesia.

## Methods

### Data source

This study utilized raw data of the 2017 Nutrition Status Monitoring Survey. This survey was a national survey using a multi-stage cluster random sampling method and was conducted by the Directorate of Community Nutrition of the Indonesian Ministry of Health [10]. The survey population was all children under 5 years old or 7–59 months in Indonesia, and there were 53,528 babies as the samples.

### Data analysis

Mother’s education level was to which degree a mother has ever completed an education. It has five categories, such as not attending school, primary school, junior high school, senior high school, and tertiary education. Another variable was mother’s employment status, which defines a type of mother’s work or employment.

The dependent variable in this study was exclusive breastfeeding practice. Seven independent variables were divided into 3 groups. The first variable was the characteristics of mothers (education level, age, marital status, employment status). The second variable was the characteristics of the children under five (age and gender). Finally, the third was a residence (urban-rural).

The Chi-Square test was utilized to test the dichotomous variables, while continuous variables were tested using the T-test. This statistical test assessed whether there was a statistically significant relationship between mother’s education level variable and another variable. Besides, a binary logistic regression test was performed at the final stage to identify disparities in the contribution of mother’s education levels to EBF practices in Indonesia. All data analyses were performed in SPSS 22 version software.

## Results

**Fig. 1:**
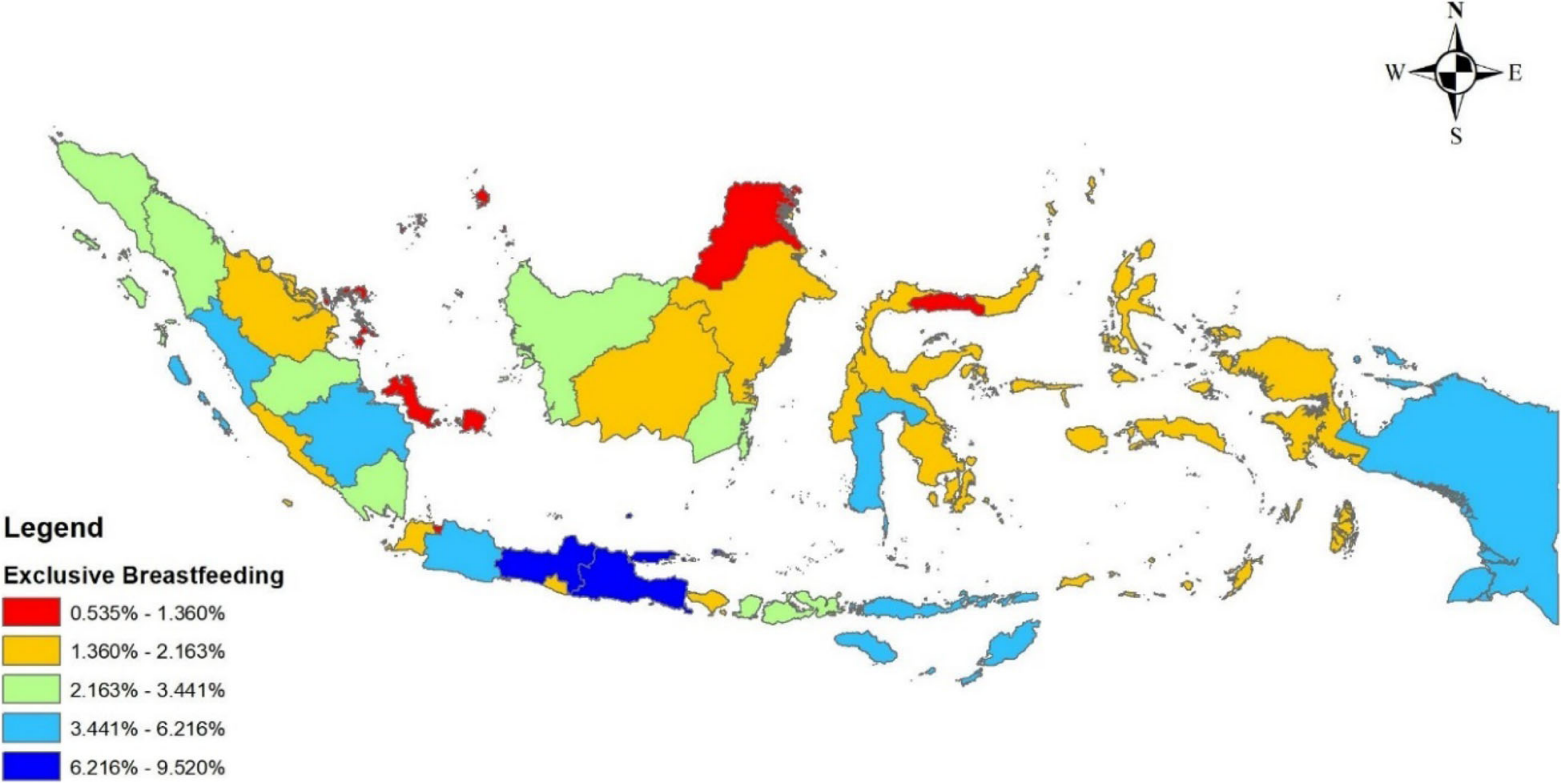
Distribution of EBF among under five (7–59 months) by provinces in Indonesia, the IDHS 2017. Source: The map depicted in the image belongs to the author

Figure 1 shows the distribution of breastfed children under five by Indonesian provinces. It indicates the largest coverage of EBF in Java.

### Descriptive statistics

Prior to a binary logistic regression test, a co-linearity test was carried out. The results of co-linearity test show that there was no co-linearity between dependent and independent variables. Table 1 shows that the tolerance value of all variables was greater than 0.10, and the VIF value for all variables was less than 10.00. Based on the result of multicollinearity test, there were no symptoms of multicollinearity in the regression model.

**Table 1:** Results for a co-linearity test of EBF in Indonesia (*n* = 53,528)

Variables	Collinearity Statistics	
	Tolerance	VIF
Mother’s Characteristics		
Education level	0.958	1.044
Age (in years; mean)	0.978	1.022
Marital status	0.997	1.003
Employment Status	0.980	1.020
Under five’s Characteristic		
Under five’s Age (in months; mean)	0.997	1.003
Under five’s Gender	1.000	1.000
Residence	0.969	1.032

*Dependent Variable: EBF status

Table 2 explains the statistical description of mother’s education level. The table points out mothers in all categories of education level mostly did not breastfeed their children. It means the practice of EBF in all education categories was relatively low.

**Table 2:** Descriptive Statistics of Mother’s Education Level and Related Variables (n = 53,528)

Variables	Mother’s Education level					All	P
	No School	Primary School	Junior High School	Senior High School	College		
EBF status							0.013*
● EBF	367 (29.84%)	4063 (34.12%)	4066 (34.49%)	8180 (34.15%)	1632 (35.13%)	18,308 (34.20%)	
● Not EBF (Ref.)	863 (70.16%)	7846 (65.88%)	7723 (65.51%)	15,774 (65.85%)	3014 (64.87%)	35,220 (65.80%)	
Mother’s Characteristics							
Age (in years; mean)	1230 (28.48)	11,909 (31.07)	11,789 (29.07)	23,594 (28.50)	4646 (30.06)	53,528 (29.33)	≤ 0.001*
Marital status							≤ 0.001*
● Single (Ref.)	5 (0.41%)	53 (0.45%)	46 (0.39%)	82 (0.34%)	18 (0.39%)	204 (0.38%)	
● Married	1208 (98.21%)	11,719 (98.40%)	11,622 (98.58%)	23,731 (99.07%)	4606 (99.14%)	52,886 (98.80%)	
● Divorce	17 (1.38%)	137 (1.16%)	121 (1.03%)	141 (0.59%)	22 (0.47%)	438 (0.82%)	
Employment Status							≤ 0.001*
● Unemployed (Ref.)	617 (50.16%)	9077 (76.22%)	9323 (79.08%)	19,867 (82.94%)	1602 (34.48%)	40,486 (75.64%)	
● Employed	613 (49.84%)	2832 (23.78%)	2466 (20.92%)	4087 (17.06%)	3044 (65.52%)	13,042 (24.36%)	
Under five’s Characteristic							
Age (in months; mean)	1230 (14.66)	11,909 (14.81)	11,789 (14.76)	23,594 (14.53)	4646 (14.69)	53,528 (14.66)	≤ 0.001*
Gender							0.605
● Male	641 (52.11%)	5987 (50.27%)	5993 (50.84%)	12,180 (50.85%)	2367 (50.95%)	27,168 (50.75%)	
● Female (Ref.)	589 (47.89%)	5922 (49.73%)	5793 (49.14%)	11,771 (49.14%)	2279 (49.05%)	26,354 (49.23%)	
Residence							≤ 0.001*
● Urban	167 (13.58%)	1989 (16.70%)	2392 (20.29%)	7559 (31.56%)	1583 (34.07%)	13,690 (25.58%)	
● Rural (Ref.)	1063 (86.42%)	9920 (83.30%)	9397 (79.71%)	16,395 (68.44%)	3063 (65.93%)	39,838 (74.42%)	

Note: Chi-Square used for dichotomous variables and the T-test used for continuous variables. *Significant at the 95% level

In Table 2, mothers who had primary education record are slightly older than mothers with other education levels. The majority of the mothers were married, but most mothers in four education levels were unemployed. Only mothers with tertiary education record were dominantly employed (65.52%).

Table 2 informs that there was a significant relationship between education levels and babies’ age. Based on babies’ gender, male babies were dominant in all education categories. However, the test results stated that the relationship between mother’s education level and babies’ gender was not statistically significant. This study also reveals most mothers lived in rural areas. This variable was proven statistically significant to affect the practice of EBF.

### Multivariate regression analysis

Table 3 shows the results of the binary logistic regression test that used “No EBF” as a reference.

**Table 3:** Results of Binary Logistic Regression (n = 53,528)

Predictors	Sig.	EBF		
		OR	Lower Bound	Upper Bound
Mother’s Characteristics				
Education level: no school	–	–	–	–
Education level: primary school	0.019^a^	1.167	1.026	1.328
Education level: junior high school	0.005^a^	1.203	1.057	1.368
Education level: senior high school	0.011^a^	1.177	1.037	1.335
Education level: college	0.003^a^	1.231	1.073	1.411
Age	≤ 0.001^a^	1.009	1.006	1.012
Marital Status: single	–	–	–	–
Marital Status: married	0.561	1.092	0.812	1.468
Marital Status: divorced	0.817	0.959	0.670	1.371
Employment status: unemployed	–	–	–	–
Employment status: employed	0.038^a^	0.954	0.913	0.997
Toddler’s Characteristic				
Age	≤ 0.001^a^	1.022	1.018	1.026
Residence				
Area: Urban	≤ 0.001^a^	1.138	1.092	1.186
Area: Rural	–	–	–	–

Note: The reference EBF status category was “Not EBF”; confidence interval of 95% for OR; ^a^significant at 95% level

It informs that all three groups of variables significantly affected the practice of EBF. Those affecting variables were mother’s age, mother’s education level, mother’s employment status, babies’ age, and residence.

This study finds mothers who graduated from primary school were 1.167 times more likely to perform EBF compared to mothers without education records (OR 1.167; 95% CI 1.026–1.328). Those who graduated from junior high school had 1.203 times possibilities to perform EBF compared to those who never attended schools (OR 1.203; 95% CI 1.057–1.368). Mothers who are high school graduates were 1.177 times more likely to perform EBF than mothers without formal education experience (OR 1.177; 95% CI 1.037–1.335). Additionally, mothers with tertiary education record had 1.203 times possibilities to give EBF than mothers without formal education record (OR 1.231; 95% CI 1.073–1.411).

Children under five whose mothers were employed had 0.954 times more likely to be breastfed those whose mothers were unemployed (OR 0.954; 95% CI 0.913–0.997). Table 3 also shows that children under five in urban areas had 1.138 times possibilities to perform EBF than those in rural areas (OR 1.138; 95% CI 1.092–1.186).

## Discussion

This study discovers that mother’s education affected the practice of EBF in Indonesia. A better education tends to give mothers more possibilities of EBF. This finding is in line with findings of other studies [16, 17]. The surveys to postnatal mothers in Nigeria and China show better education positively contributed to the breastfeeding process and the rate level of EBF [16, 17]. Some studies in America added self-efficacy as a variable to find the correlation of maternal education and the practice of EBF. Higher education levels were correlated with better self-efficacy scores. Mother’s education level had a positive relationship with the practice of EBF [8, 9].

Similarly, another study in European multiregions finds that mothers who were younger and less educated were more likely to stop breastfeeding before their babies were aged 6 months. In other words, they did not perform complete EBF. Education level, parity, and socioeconomic factors could indicate whether mothers breastfeed or not their babies [18]. Some studies in Chile also found psychosocial factors, such as maternal IQ and low-risk prenatal behavior at birth, could affect the duration of breastfeeding [19, 20]. Another study conducted in Eastern Indonesia, which analyzed data of the 2012 Indonesian Family Life Survey, concluded the same thing. This study took a smaller sample size (1138 under five) and found that mother’s education levels had a positive effect on the success rates of EBF [21].

Moreover, this study uncovers unemployed mothers had better EBF practice. This result is similar to the results of other studies indicating employed mothers were less likely to perform EBF. They tend to have less time and opportunity to interact with children, including giving breastfeed to their children [21, 22]. Studies in Qatar and Ethiopia find that the employment status was one of the barriers to the success of EBF. Plausibly mothers must return to work immediately after the maternity leave period runs out [23, 24]. Having flexible work schedules and workplace proximity to home can assist the sustainability of breastfeeding [25].

Another study carried out in Vietnam indicates that teamwork parenting could increase the success of EBF. In this study, husband’s role was also observed to be one of the predictors of EBF success [26]. Supporting this fact, RCT research in Canada finds collaboration between parents influenced knowledge, perceptions, practices, and duration of EBF [27]. In India, social support became one of the determinants of EBF success [1].

This present study, furthermore, shows that children under five in urban areas had a better EBF intake. This is probably due to better information exposure about the benefits of EBF in urban areas than in rural areas. The effect of information exposure that may improve mother’s knowledge about EBF was proven as one determinant of EBF success by several studies [17, 21, 28, 29]. In Indonesia, urban areas tend to have better access to health services and information than rural areas, and this leads to higher coverage of health programs [30, 31].

To end this discussion, this study also suggests the government to formulate policies focusing on clear targets to be achieved by referring to these study findings. It can target mothers who have poor education, are employed, and live in rural areas. Policies focusing on these targets need to be implemented for a wider coverage of EBF.

### Limitation

The study utilized big data of the 2017 IDHS survey. The data are not presenting facts deeply so that the findings in this study have not captured different phenomena in the number of indigenous Indonesians, who have their local wisdom of breastfeeding practices. Mother’s cultural background can be an adequate differentiator [12, 14, 32]. Besides, the study also did not involve the participation of parents, parents-in-law, and family to indicate the practice of EBF. While, previous studies find this variable could affect EBF [32, 33]. It is necessary to conduct further qualitative studies to capture or deepen some phenomena in question.

## Conclusions

It could be concluded that there was a positive effect of mother’s education on the practice of EBF in Indonesia. Other variables, such as mother’s age, mother’s employment status, babies’ age, and residence were some predictors of EBF success rates in Indonesia.

The government needs to issue policies that focus on specific targets as identified in this study. It can target mothers who have poor education, are employed, and live in rural areas to expand the coverage of EBF in Indonesia.

## Data Availability

The 2017 Nutrition Status Monitoring Survey data used to support these findings of this study were supplied by the Directorate of Community Nutrition of the Indonesian Ministry of Health under license and so can not be made freely available. Requests for access to these data should be made to the Directorate of Community Nutrition of the Indonesian Ministry of Health.
